# Intervening at the Setting Level to Prevent Behavioral Incidents in Residential Child Care: Efficacy of the CARE Program Model

**DOI:** 10.1007/s11121-016-0649-0

**Published:** 2016-05-03

**Authors:** Charles V. Izzo, Elliott G. Smith, Martha J. Holden, Catherine I. Norton, Michael A. Nunno, Deborah E. Sellers

**Affiliations:** Bronfenbrenner Center for Translational Research (BCTR), Cornell University, Beebe Hall, Ithaca, NY 14853 USA

**Keywords:** Behavioral incidents, Child welfare, Children, Evidence-based social work, Institutional care, Organizational change, Residential care, Therapeutic milieu

## Abstract

The current study examined the impact of a setting-level intervention on the prevention of aggressive or dangerous behavioral incidents involving youth living in group care environments. Eleven group care agencies implemented Children and Residential Experiences (CARE), a principle-based program that helps agencies use a set of evidence-informed principles to guide programming and enrich the relational dynamics throughout the agency. All agencies served mostly youth referred from child welfare. The 3-year implementation of CARE involved intensive agency-wide training and on-site consultation to agency leaders and managers around supporting and facilitating day-to-day application of the principles in both childcare and staff management arenas. Agencies provided data over 48 months on the monthly frequency of behavioral incidents most related to program objectives. Using multiple baseline interrupted time series analysis to assess program effects, we tested whether trends during the program implementation period declined significantly compared to the 12 months before implementation. Results showed significant program effects on incidents involving youth aggression toward adult staff, property destruction, and running away. Effects on aggression toward peers and self-harm were also found but were less consistent. Staff ratings of positive organizational social context (OSC) predicted fewer incidents, but there was no clear relationship between OSC and observed program effects. Findings support the potential efficacy of the CARE model and illustrate that intervening “upstream” at the setting level may help to prevent coercive caregiving patterns and increase opportunities for healthy social interactions.

In the USA, there were about 56,000 youth living in group care in 2013 (US Department of Health and Human Services 2014). Group care agencies have seen an increase in the proportion of youth with complex emotional and behavioral problems (Duppong-Hurley et al. 2009), most of whom are involved with the child welfare and/or mental health service systems (James et al. 2006). There is growing recognition of the imperative to advance the healthy social and emotional development of these young people, rather than focusing solely on safety and permanency outcomes (Samuels 2012). Yet, many group care providers are not adequately prepared or supported to meet the developmental and relational needs of the youth they serve (Colton and Roberts 2007; Hoagwood et al. 2001; Kakuma et al. 2011). With little training in child development, behavior management, or dealing with complex trauma, they are less able to help residents manage emotions and more likely to respond to distressed youth in ways that trigger dangerous behavioral incidents (e.g., aggression, self-harm, running away). Given the serious risk profile faced by these youth, prevention can play a key role by enriching the social environment and reducing their exposure to traumatic or dangerous experiences that can further impede development (Anda et al. 2006).

Prevention efforts can be enhanced by focusing on contextual factors that influence behavioral outcomes and provider practices (Biglan 2004). Aspects of organizational social context can create a set of shared expectations and beliefs that affect how providers work with consumers in children’s services organizations (Glisson et al. 2008). These can either facilitate or stifle attempts to adopt evidence-based practices and innovations (Aarons and Sawitzky 2006).

Few setting-level interventions have reported evidence to demonstrate positive outcomes within group care settings. The Sanctuary® Model works at both clinical and organizational levels to create a trauma-informed community. A quasi-experimental study of Sanctuary reported significant effects on coping skills and on aspects of the therapeutic program environment (Rivard et al. 2005). The Teaching Family Model (TFM) promotes the use of behavior modification strategies within the broader context of ecological and systemic change. Multiple studies have demonstrated positive impact on outcomes such as school achievement (Ringle et al. 2010), illegal behavior (Kirigin et al. 1982), and behavioral incidents (Duppong-Hurley et al. 2006). The Attachment, Self-Regulation, and Competency (ARC) framework guides trauma-informed professional practice across 10 skill areas. Application of ARC in a residential treatment setting has shown improvements in post-traumatic stress symptoms, problem behavior, and use of physical restraint (Kinniburgh et al. 2005). Given the promising but sparse knowledge base on setting-level interventions in group care, leading voices have called for more research to identify models that seek to foster positive social and emotional functioning by producing a healthy milieu (James 2014; Whittaker 2004).

In the current study, we examined the impact of Children and Residential Experiences (CARE; Holden 2009), a principle-based program designed to enhance the social dynamics in group care settings through targeted staff development and ongoing reflective practice (i.e., learning through focused attention to one’s own practice). CARE explicitly uses an ecological approach to help agencies transition from simply maintaining compliance to creating a living environment that offers/provides developmentally enriching experiences and a “sense of normality” (Anglin 2002). CARE offers a set of practical heuristics or principles to guide practice and then establishes, trains, and supports a local implementation team to facilitate broad application of the principles to build congruency throughout the agency. CARE consultants follow a standardized set of steps to train and support staff, but unlike other programs of its kind, local implementation is determined by agency leaders and staff using their own creativity and professional judgment. This approach cultivates personal investment and ownership and serves to reduce the sense of being constrained or controlled that is often elicited by more directive program models (Borntrager et al. 2015).

We assessed whether CARE implementation led to fewer documented reports of behavioral incidents. Incident reports are not simply indicators of behavioral dysregulation but also represent markers for a broader pattern of interaction among youth and adults. For example, staff that understand and respond with more sensitivity to emotionally distressed residents will be better able to diffuse momentary situations that would otherwise culminate in dangerous or destructive behaviors that require formal documentation. Similarly, by learning to set developmentally appropriate expectations, staff will create fewer opportunities for youth to feel frustration or anger and more opportunities for success. We tested the following hypotheses: (1) Time trends in the rate of behavioral incidents will decrease during the implementation period relative to baseline, indicating beneficial program effects; (2) more positive organizational social context (OSC) will be associated with lower levels of behavioral incidents; and (3) program effects will be greater in agencies with a more positive OSC.

## Method

### Research Design

The multiple baseline interrupted time series design (ITS) is recommended for evaluating interventions when randomized controlled trials are not practical (Biglan et al. 2000; Shadish et al. 2002). ITS was used to examine how CARE implementation was related to changes in the frequency of behavioral incidents in two successive cohorts of agencies. Specifically, we compared incident rates in the 12 months before implementation (baseline period) to rates in the 36 months during implementation (implementation period).

Attributing a causal inference to the program effect is strengthened if key threats to internal validity are addressed, including maturation, history, and selection (Shadish et al. 2002). Comparing the baseline and implementation periods helps to rule out maturation (i.e., continuation of an existing trend) as an alternative explanation. We examined potential history effects by comparing the five agencies implementing in 2010 (cohort 1) with a comparable group of six agencies that began implementation in 2011 (cohort 2). If program effects are found in both cohorts, historical factors become less likely as an alternative explanation. We also reduced potential selection effects by including multiple agencies from across the state.

### Participating Agencies

Data from 11 agencies were included in this study. Agencies were recruited in several ways, including presentations to a statewide association of group care agencies, letters sent directly to eligible agencies, and through word of mouth. Criteria for participation included serving primarily youth referred by social services, having not already been exposed to CARE, willingness to be placed on a 12-month waitlist if needed, and being licensed by a state agency. Although typically conducted through fee-for-service contracts (http://rccp.cornell.edu/care/care_main.html), services were provided free of charge in the current study. Sixteen agencies committed to participate, seven of which were part of a larger parent organization with campuses across the state. These seven agencies participated as separate entities, each with its own leadership structure, and were distributed among both cohorts. Among the 16 agencies, one became ineligible due to a change in target population, one closed before implementation began, one discontinued due to change in administrative priorities, and two were excluded from analyses because their database did not record information about specific incident types. The remaining 11 agencies were included in the study. At the start of CARE, the average number of residential staff at these agencies was 13 [min = 4; max = 23], and the average number of youth was 24 [min = 9; max = 44], resulting in an average youth to staff ratio of 1.81 [min = 0.82; max = 2.75]. Most agencies served males and females typically from 7 to 18 years of age. One agency served only males and followed a camp model maintaining up to four outdoor campsites year-round. All agency caregivers lived full time in the home for 1–2-week shifts. Three agencies allowed only married caregivers, and eight agencies typically hired unrelated caregivers. All agencies previously relied on homegrown systems of rules and consequences and an assortment of enrichment activities (e.g., recreation, life skills training) but had no coherent model that guided day-to-day childcare and management matters. No agency had received systematic training with a specific evidence-based practice or treatment approach.

### Program Description and Implementation

CARE engages residential childcare agencies in a systematic effort to reorient their practices around a set of six evidence-informed principles. A pair of CARE Consultants works with each agency for 3 years to support programming that is (1) relationship-based (i.e., helps youth form healthy models of adult-child relationships and builds their capacity for healthier relationships in the future), (2) trauma-informed (i.e., sensitive to youth’s trauma history), (3) developmentally focused (i.e., provides more opportunity for normative developmental experiences and adapts expectations to meet the unique needs of youth), (4) family-involved (i.e., seeks to understand and adapt to families’ cultural norms and tries to promote active family involvement), (5) competence-centered (i.e., creates opportunities for building self-efficacy and competence for dealing with life circumstances), and (6) ecologically oriented (i.e., enriches the physical and social environment to create a therapeutic setting). For most agencies, this process calls for changes in theoretical perspective, organizational norms, and role expectations. All CARE Consultants have graduate degrees in Social Work or related fields and several years of leadership and supervisory experience in group care settings.

An essential implementation activity is the development of a CARE Implementation Team (IT) that includes agency leadership, supervisors, and key training and clinical staff. Its role involves providing support, modeling, and mentoring to staff as they incorporate CARE principles into their work. The team also builds structures and processes that facilitate application of the CARE principles and their eventual integration into the agency culture.

The leadership and ITs are trained in the CARE principles through a 5-day manualized program, and a group of agency-based trainers are prepared to deliver the same 5-day training to remaining staff. CARE Consultants provided quarterly on-site technical assistance (TA) visits to implementation teams and other agency staff. TA activities involve observation and feedback, training and coaching for front-line supervisors, developing routines for reflective practice, and addressing organizational barriers to creating a more therapeutic milieu.

### Data Collection

In the month prior to program onset, agency personnel completed an anonymous survey with questions about demographics and their perceptions about organizational climate and culture. Respondents were informed that survey data would not be linked to their identity and that no agency personnel would see them.

Based on licensing and accreditation guidelines, standard agency policies require that staff complete a written report for any incident involving substantial risk of danger to a resident or staff member. Agencies provided monthly behavioral incident data from their administrative records during the baseline and implementation periods. Each year, agency quality assurance staff were asked to count the number of incident reports filed in the previous year, indicating the monthly frequencies for each of five incident types: verbal threats or physical aggression toward staff, verbal threats or physical aggression toward peers, an act or threat of self-harm, property destruction, and attempted or completed runaways. Staff did not record incident severity, but all incidents were sufficiently dangerous or destructive to require a written report. Incident counts were provided only at the agency level (i.e., we received no information about incident frequency within each residential unit). Incidents involving multiple residents were counted separately for each resident, unless the resident was only a victim in the incident. Agencies unable to dedicate staff for this job provided full incident reports with identifying information redacted. With approval from the Cornell University IRB, the research team reviewed reports and completed the incident record form.

We examined potential threats to measurement validity in time series data identified by Shadish et al. (2002). Although recording procedures varied across agencies, there were only minor changes across time within agencies. Agency representatives indicated that there were no changes in the types of incidents requiring a formal report, the expectations or incentives to complete reports, or the definitions for any incident type. Notably, CARE implementation did not address behavioral incident reporting or the conditions that should trigger a report. Therefore, the dependent variable was not confounded with the program content.

In seven cases, agencies changed to a new management information system after the first 1–2 years of data collection but reported that this had no effect on whether reports were submitted. Our data collection process remained consistent except for one agency that assigned a staff member to complete our incident record form for half the study period but provided incident reports for our team to code for the other half.

### Measures

#### Behavioral Incidents

For each agency, the number of incidents for each of the five incident types, aggression toward staff, aggression toward peers, self-harm, property destruction, and runaways, was recorded for each month. The average number of residents for the month was used to compute per capita rates.

#### Organizational Social Context

The baseline staff assessment included the OSC survey, which assesses dimensions of culture (proficiency, resistance, rigidity) and climate (stress, engagement, functionality) at the agency level (Glisson and Hemmelgarn 1998). Glisson et al. (2013) used latent profile analysis with a national sample of 100 child mental health clinics to derive three profiles (1 = negative, 2 = average, 3 = positive) based on the pattern of scores across the six subscales. Using estimates from the national sample, profile scores were computed for agencies in our study representing the probability of membership in one of the three classes. Specifically, negative profiles reflect lower scores on engagement, functionality, and proficiency and higher scores on stress, resistance, and rigidity. Positive profiles reflect an opposite pattern of subscale scores.

#### Implementation Progress

*Consultant-level implementation* involves the training and support to agencies provided by CARE Consultants and was documented by recording training and visit dates and the number of staff participating. *Local implementation progress* (LIP) reflected agency-level efforts to use the CARE principles to critically review and modify their own practices with regard to childcare, programming, and staff management. Based on their experience during the third year, the CARE consultants most familiar and engaged with each agency independently provided retrospective LIP ratings using a five-point scale: 1 = “no progress,” 2 = “planning,” 3 = “sporadic progress,” 4 = “consistent progress,” and 5 = “fully achieved/exemplary.” Ratings were made on the following seven dimensions: sustained CARE leadership support, CARE reflected in formal policies, capacity for training and professional development, maintaining an effective implementation team, deliberate efforts to integrate CARE principles across multiple levels of the agency, quality of interactions between staff and youth, and quality of interactions among staff. These dimensions reflect elements of effective implementation identified by an advisory committee composed of agency directors who have successfully implemented CARE. A composite LIP score was computed as the mean of all seven ratings. For agencies rated by two consultants, reliability was high (intraclass correlation = 0.78, 95 % CI [0.61, 0.88]), and the two ratings were averaged. Four agencies were rated by one consultant.

### Data Analysis

For each of the five types of behavioral incidents, we constructed a mixed effects negative binomial regression model to estimate the number of behavioral incidents per resident per month. Negative binomial regression is appropriate for low-frequency count data that are positively skewed (Long 1997; Osgood 2000), and including mixed effects accounts for the clustering of the time series data within agencies (Rabe-Hesketh and Skrondal 2012). Each model contained the same parameters, which are listed in Table 2. Fixed effects were modeled by estimating an intercept for the frequency of incidents in the month just prior to the start of CARE (month 0) and two slopes, or time trends: one for the baseline period prior to CARE (months −12 to 0) and one for the program implementation period (months 1 to 36). Three covariates were included to adjust the intercept: (1) an indicator for the cohort to which the agency belonged, (2) an indicator for whether the agency was among a set of agencies that shared the same parent organization, and (3) the OSC profile score at month 0. Trend line covariates for cohort and OSC profile score were added to test for moderation in slopes during the baseline and implementation periods. An exposure variable, ln(Residents), was included so that the estimated counts of incidents could be interpreted as per capita rates.

For the random effects portion of the model, we accounted for agency-level variation in the mean level of incidents and in the trends over time that was not captured by the fixed effects parameters. Finally, a dispersion parameter was included to account for variance beyond that allowed by a Poisson distribution.

## Results

### Baseline Period Characteristics

Summary statistics for the months during the baseline period are provided in Table 1. The average number of care days per month was 753 and ranged from 217 to 1583. Converting care days to residents, the average number of residents per month was 25, ranging across agencies from 7 to 51. On average, the monthly incident rate was 0.26 per resident or just over one incident per month for every four residents. Monthly rates varied widely during baseline from 0 to 4.25. Aggression toward peers was most common, followed by aggression toward staff and runaway. Property destruction and self-harm were the least frequent incidents. Table 1 also summarizes OSC profile scores and the subscale *t* scores from which they were derived. The *t* scores are based on a national sample of mental health service agencies (Glisson et al. 2008). At baseline, six agencies scored in the average range, two were in the negative range, and three were in the positive range.

**Table 1:** Unadjusted monthly summary statistics during the pre-CARE baseline period

Variable	*M* (SD)	Minimum	Maximum	Cohort difference
Care days	753.01 (356.12)	217	1583	[8.49, 257.11]*
Residents	24.75 (11.62)	7	51	[0.30, 8.41]*
Incidents per resident				
All incidents	0.26 (0.46)	0.00	4.25	[−0.14, 0.19]
Aggression toward peers	0.10 (0.22)	0.00	2.13	[−0.07, 0.09]
Aggression toward staff	0.06 (0.15)	0.00	1.38	[−0.05, 0.06]
Runaway	0.05 (0.09)	0.00	0.64	[−0.03, 0.04]
Property destruction	0.03 (0.05)	0.00	0.26	[−0.02, 0.01]
Self-harm	0.03 (0.05)	0.00	0.25	[0.00, 0.04]*
OSC latent profile score	2.08 (0.65)	1.00	3.00	[−0.05, 0.39]
OSC culture				
Proficiency	52.32 (12.35)	19.18	64.54	[−1.17, 6.61]
Rigidity	59.14 (5.28)	50.35	66.50	[−4.90, −1.55]**
Resistance	58.69 (7.86)	47.84	74.88	[−3.55, 1.59]
OSC climate				
Engagement	50.61 (9.69)	33.27	65.98	[4.04, 10.24]**
Functionality	57.11 (10.50)	34.91	72.15	[1.16, 7.96]**
Stress	47.47 (6.24)	39.51	60.60	[−4.26, 0.01]

Cohort difference shows the 95 % confidence interval for the cohort 1–cohort 2 *t* test.

*M* mean, *SD* standard deviation

*p < 0.05, **p < 0.01

Two-sample *t* tests of unadjusted means indicated that cohorts 1 and 2 were equivalent at baseline with regard to four of the five incident types as well as the overall frequency of incidents. Baseline cohort differences were found for self-harm, number of care days, and three OSC subscales. Inclusion of resident census and OSC profile in our regression models adjusted for these baseline cohort differences.

### Program Implementation

There was little variance in consultant-level implementation activities. All agencies established an implementation team that remained intact throughout the study period. Training sessions conducted in year 1 reached an average of 78 % of agency staff (range 68 to 88 %). Consultants trained at least one local CARE educator at each agency within the first year, and additional educators were trained as needed to continue training new staff and provide refreshers. All agencies participated in 12 quarterly visits over the 36-month implementation period, lasting from 1 to 5 days depending on mutually determined need. Consistent with the CARE design, the types of agency-led implementation activities varied across agencies, taking different forms depending on local needs and priorities. In the third year of intervention, LIP ratings averaged across the seven dimensions indicated that six agencies had made “consistent” or “exemplary” progress and four agencies made “sporadic” progress. One agency was still considered to be in the planning stage and had made only minor progress, a rating of 2.

There was no simple relationship between OSC profile and LIP ratings. The three agencies with positive profiles, ranging from 2.8 to 3.0, showed only sporadic progress, while the two in the negative range (1.1 to 1.3) showed consistent progress. The six agencies with average profiles (1.7 to 2.3) were distributed evenly across the range of LIP ratings.

### Interrupted Time Series Regression Models

Results of the ITS regression models are presented in Table 2. Model parameters are reported in the form of incidence rate ratios. Fixed effects are reported in the upper portion, showing the rate of incidents at the start of CARE and the time trends during the baseline and implementation periods. Random effects appear in the lower portion. Significant variance between agencies was found in the frequency of incidents at the start of CARE and, to a lesser extent, in the trends over time.

**Table 2:** Incidence rate ratios and 95 % confidence intervals for mixed effects negative binomial regression models

	Aggression toward peers	Aggression toward staff	Property destruction	Self-harm	Runaway
Fixed effects					
Estimates at the start of CARE					
Incidents per resident, Cohort 1^b^	0.22 [0.14, 0.34]**	0.10 [0.04, 0.26]**	0.05 [0.03, 0.10]**	0.05 [0.02, 0.10]**	0.04 [0.02, 0.11]**
Cohort difference^a^	0.62 [0.34, 1.13]	0.65 [0.18, 2.38]	1.20 [0.51, 2.83]	0.34 [0.11, 1.03]	1.07 [0.30, 3.83]
Parent organization difference^a^	0.60 [0.36, 0.98]*	0.43 [0.12, 1.59]	0.28 [0.12, 0.62]**	1.03 [0.36, 2.98]	1.19 [0.35, 4.11]
OSC profile, centered at 2.0^a^	0.31 [0.19, 0.51]**	0.39 [0.15, 1.06]	0.29 [0.13, 0.62]**	0.73 [0.30, 1.77]	0.56 [0.22, 1.45]
Baseline period trend, cohort 1	1.18 [1.10, 1.27]**	1.19 [1.07, 1.32]**	1.16 [1.04, 1.28]**	1.08 [0.99, 1.17]	1.03 [0.95, 1.12]
Cohort moderation^a^	0.86 [0.78, 0.95]**	0.85 [0.74, 0.98]*	0.92 [0.81, 1.06]	0.94 [0.83, 1.07]	1.02 [0.92, 1.14]
OSC profile moderation^a^	0.85 [0.79, 0.92]**	0.98 [0.87, 1.10]	0.93 [0.83, 1.05]	0.98 [0.88, 1.10]	1.02 [0.94, 1.10]
Implementation period trend, cohort 1	0.94 [0.91, 0.97]**	0.93 [0.89, 0.97]*	0.95 [0.91, 1.00]*	0.92 [0.88, 0.97]**	0.96 [0.94, 0.98]**
Cohort moderation^a^	1.06 [1.01, 1.10]*	1.04 [0.96, 1.11]	1.00 [0.94, 1.07]	1.07 [1.01, 1.13]*	1.02 [0.99, 1.05]
OSC profile moderation^a^	1.02 [0.99, 1.06]	1.01 [0.95, 1.07]	1.02 [0.97, 1.07]	1.01 [0.97, 1.06]	1.03 [1.01, 1.06]**
Random effects					
Agency-level variance					
At the start of CARE	0.07 [0.01, 0.35]	0.76 [0.25, 2.28]	0.19 [0.03, 1.26]	0.50 [0.15, 1.69]	0.79 [0.31, 2.02]
Trends over time	0.001 [0.000, 0.002]	0.003 [0.001, 0.007]	0.002 [0.000, 0.006]	0.001 [0.000, 0.005]	0.000 [0.000, 0.000]
Dispersion parameter, ln(alpha)^b^	0.19 [−0.03, 0.40]	0.56 [0.30, 0.82]**	0.33 [−0.02, 0.69]	0.12 [−0.31, 0.54]	0.32 [0.03, 0.62]*

Incidence rate ratios are reported with the 95 % CI shown in brackets. An additional parameter, ln(Residents), was included as the exposure variable in order to convert the counts to per capita rates. The model intercept is labeled as *Incidents per resident, cohort 1*. Because cohort 1 agencies served as the reference group, *Cohort moderation* and *OSC profile moderation* indicate the degree to which the trends differed as a function of cohort or OSC profile score.

*CI* confidence interval, *OSC* organizational social context

*p < 0.05, **p < 0.01

^a^ *p* values were computed for difference from 1

^b^ *p* values were computed for difference from 0

#### Estimate at the Start of CARE

Per capita rates at month 0 were greater than zero for all incident types, and no cohort differences were found. Having a parent organization and having a positive OSC profile were associated with substantially lower rates of aggression toward peers and property destruction.

The presence of consistent seasonality effects were assessed by adding calendar month to the a priori model. The overall contrast was not significant for any of the outcomes, so calendar month was not included as a covariate in the final models.

#### Baseline Trend

There was an increasing trend for aggression toward peers, aggression toward staff, and property destruction during baseline for cohort 1, which occurred in 2009. In cohort 2, for whom the baseline period was in 2010, an increasing trend was evident for property destruction, and other incident types showed no change. More negative OSC profiles were associated with a steeper baseline increase in aggression toward peers. OSC profile was unrelated to the baseline trends for other incident types.

#### Implementation Trend and Program Effect Estimates

During CARE implementation, significant decreases in incident rates of 4 to 8 % per month were observed for all outcomes in cohort 1. For cohort 2, the findings were less consistent. Cohort 2 trends did not differ from cohort 1 for sggression toward staff, property destruction, and runaway, but they did differ for aggression toward peers and self-harm. OSC profile moderated the implementation trend for runaway incidents with more negative OSC profile associated with greater improvement during the implementation period. Given that the runaway rate at baseline was already quite low for agencies with positive OSC profiles (0.03, on average), the lack of a trend is likely the result of a floor effect.

Table 3 presents trend estimates for the baseline and implementation periods and the difference between periods, labeled as the program effect. We constructed linear combinations of the model parameters and ran planned contrasts (StataCorp 2013). Results are averaged across cohort for three outcomes, but they are presented separately for the two outcomes when we observed that cohort moderated the implementation trend. For aggression toward staff, property destruction, and runaway, there was a declining trend during implementation, and it was significantly different from the baseline trend, as predicted. This same pattern held for aggression toward peers and self-harm but was limited to cohort 1.

**Table 3:** Adjusted estimates for trends and program effect

Incident type	Baseline trend	Implementation trend	Program effect
Aggression toward staff	1.10** [1.03, 1.18]	0.95** [0.92, 0.99]	0.87** [0.81, 0.94]
Property destruction	1.11** [1.04, 1.19]	0.95** [0.92, 0.98]	0.86** [0.79, 0.92]
Aggression toward peers	1.09** [1.04, 1.05]	0.96** [0.94, 0.98]	0.89** [0.84, 0.93]
Cohort 1	1.18** [1.10, 1.27]	0.94** [0.91, 0.97]	0.79** [0.73, 0.86]
Cohort 2	1.01 [0.95, 1.08]	0.99 [0.96, 1.02]	0.98 [0.91, 1.04]
Self-harm	1.04 [0.98, 1.11]	0.95** [0.93, 0.98]	0.92* [0.85, 0.99]
Cohort 1	1.08 [0.99, 1.17]	0.92** [0.88, 0.97]	0.85** [0.78, 0.95]
Cohort 2	1.01 [0.93, 1.11]	0.99 [0.95, 1.03]	0.97 [0.88, 1.08]
Runaway	1.04 [0.99, 1.10]	0.97** [0.95, 0.99]	0.93* [0.87, 0.99]
Negative OSC	1.01 [0.91, 1.13]	0.93** [0.90, 0.96]	0.92 [0.80, 1.04]
Average OSC	1.03 [0.95, 1.12]	0.96** [0.94, 0.98]	0.93 [0.84, 1.03]
Positive OSC	1.05 [0.94, 1.18]	0.99 [0.96, 1.02]	0.94 [0.83, 1.08]

Estimates are derived from negative binomial regression models. *Baseline trend* represents the average monthly proportional change during the baseline period. *Implementation trend* represents the average monthly proportional change in incidents during program implementation. *Program effect* is the proportional comparison of the two trends. Statistics are also shown by cohort or OSC profile when a significant moderation effect was found. *p* values were computed for difference from 1.

*CI* confidence interval, *IRR* incidence rate ratio, *SE* standard error

*p < 0.05, **p < 0.01

To investigate the impact of the increasing baseline trends on program effect estimates, we ran a separate set of analyses excluding agencies that showed baseline time trends (one to four agencies, depending on the outcome). Implementation trends decreased relative to baseline for four variables: aggression toward peers (IRR = 0.90, 95 % CI [0.85, 0.97]), aggression toward staff (IRR = 0.91, 95 % CI [0.84, 0.99]), property destruction (IRR = 0.88, 95 % CI [0.79, 0.99]), and self-harm (IRR = 0.93, 95 % CI [0.86, 1.00]). There were no significant differences by cohort.

**Fig. 1:**
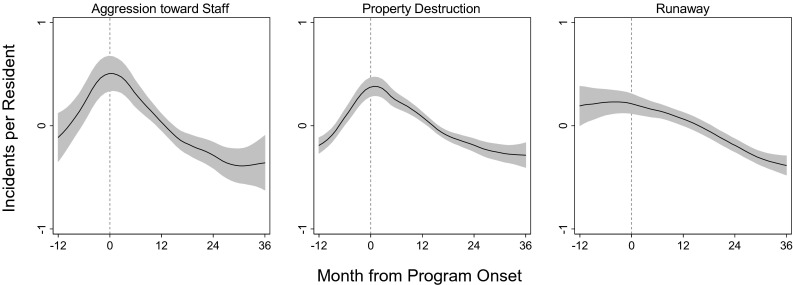
Model-adjusted monthly counts and 95 % confidence intervals for incidents per resident over the 4-year study period. Each agency’s counts have been centered at the agency mean to account for overall agency differences in the frequency of incidents. The *dashed line* represents the start of program implementation

Figure 1 illustrates model-adjusted estimates for the frequency of incidents per resident over the entire 4-year study period. The three outcomes that showed consistent results across cohorts are shown. To adjust for overall agency differences in the frequency of incidents, estimates were centered at each agency mean.

### Moderating Role of Implementation Progress

We conducted post hoc analyses to explore whether implementation progress was related to the reduction in incident rates during implementation, relative to the baseline period. A reduced version of the negative binomial regression model shown in Table 2 was constructed by removing terms related to time trend and by removing interaction terms with cohort. Observations were limited to baseline period and the final year of implementation, given that LIP ratings focused only on the final year. Finally, given the limited number of agencies, three-way interactions between CARE, LIP, and cohort were not stable. Within these reduced models, significant effects of CARE were found, as previously described. In no case, however, was LIP related to incident rates nor did it moderate the relationship between CARE and the outcomes.

## Discussion

The current study examined the effects of CARE, a setting-level intervention to improve residential care quality. The results support our first hypothesis indicating that agencies’ participation in CARE led to significant declines for three important types of behavioral incidents (aggression toward staff, property destruction, and runaways). Other programs have reported similar findings (Nunno et al. 2003; Duppong-Hurley et al. 2006), although it is notable that in one major study of the Teaching Families Model, effects were limited to alleged criminal offenses and no effects were found on runaways or unauthorized absences (Kirigin et al. 1982). Our results are less consistent for aggression toward peers and self-harm. Given that significant declines were not observed in cohort 2, we cannot rule out the possibility that the decline in cohort 1 was due to historical factors or some other conditions unique to those five agencies. The increasing baseline rates for some incident types may have contributed to the significant declines observed during implementation. However, program effects persisted after excluding agencies with significant baseline trends, making regression to the mean a less plausible explanation for our findings (Smith 2012).

Our results supported the second hypothesis, indicating that more positive OSC predicted fewer incidents involving aggression toward peers and property destruction. This is consistent with Glisson and Hemmelgarn (1998) who found that OSC predicted better service quality and well-being in children’s mental health settings. Our third hypothesis was not supported. Moderation of implementation trend by OSC profile was observed in only one variable and showed steeper incident reductions when baseline profiles were negative. The data do not support our assumption that positive OSC would facilitate better implementation; rather, agencies rated as having the most consistent progress tended to have either negative or average OSC profiles. This would be consistent with an explanation that poorer conditions at the start may make participants more motivated to try new innovation and more receptive to help. Though this pattern of findings is by no means consistent across studies (Eckenrode et al. 2003), it may have played out that way here.

There have been few rigorous studies of organizational or setting-level interventions in the field of residential youth care (James 2014). Our use of the interrupted time series design provides strong evidence to conclude that the observed improvements in agencies were at least partly due to their participation in CARE. This goes a step beyond the findings of previous studies that demonstrated important mean differences between pre- and post-intervention periods (Duppong-Hurley et al. 2006; Jones and Timbers 2002). By modeling time trends during baseline and implementation periods separately, we estimated differences in the *rate of change* between these periods, thus providing a clearer picture of the nature and timing of program effects.

Our study is strengthened further by using uniform measurement and analytic methods across 11 agencies varying in size, staff characteristics, and cultural norms. The issue of external validity is significant given the current landscape of intervention research in group care. A study of Sanctuary reported results from eight cottages within a single campus (Rivard et al. 2005), and few studies of TFM involved more than one campus (Jones and Timbers 2002; Kirigin et al. 1982). More generally, studies reporting program effects on behavioral incidents tend to include only one (Barton et al. 2009; Duppong-Hurley et al. 2006; Hodgdon et al. 2013) or two campuses (Jones and Timbers 2002).

Our research demonstrates that by focusing only at the staff and organization levels, CARE significantly reduced the prevalence of dangerous or destructive behavioral incidents that create a distressing, non-therapeutic environment in the daily lives of residents. Given that incidents such as these can escalate into physical restraint or, in extreme cases, result in injury or death (Day 2002; Nunno et al. 2006), the potential benefits of reducing behavioral incidents can be profound. Its impact can also be calculated in terms of the psychological, social, and ecological sequelae that often follow these stressful events. Finally, aggression in this setting exposes other vulnerable youth to “ambient stress” which adds to the cumulative developmental risk they already face (Evans 2003; Gorman-Smith and Tolan 1998).

CARE is explicitly designed to help staff, managers, and leadership use a set of organizing principles to support better quality child care at the agency. Our LIP assessment showed that in addition to staff training, most agencies engaged in many promising activities to improve their organizational functioning and facilitate more therapeutic interactions with youth. Within this programmatic context, it is plausible to attribute the downward trend in incident rates partly to improved youth-adult relationships and greater flexibility, such that staff were more likely to respond to transgressions in ways that avoided power struggles, hostility, or alienation. This explanation is consistent with results from Anglin’s qualitative study (Holden et al. 2014) based on interviews and observations across seven experienced CARE agencies that were actively working to sustain CARE after 4 years of implementation. Staff reported greater understanding of trauma’s effect on youth behavior, leading to fewer confrontations and power struggles, less fear, and a more peaceful environment in the homes.

Some methodological limitations should be considered when interpreting this study’s findings. First, with the exception of “runaways,” the incident report data used in our study are based on the observations and judgments of agency staff about whether each incident required a formal report. These data should not be viewed simply as a measure for the rate of behavioral dysregulation but also as a marker for a set of dynamic social interactions among residents and staff. Our data do not make distinctions based on incident severity (e.g., some peer aggression incidents were likely more severe than others), so it is unclear whether there was a differential program effect for more vs. less severe incidents. Our agency-level data also does not allow us to identify highly disruptive individuals that may enter care and affect incident trends.

Our data allow for the estimation of trends during the 36-month implementation period, but it is unknown whether these improvements continued. One multisite TFM study found that its strong initial program effects were evident only during the 12-month implementation period, but no longer evident by 12-month follow-up (Kirigin et al. 1982). This highlights the value of including extended follow-up assessments in impact studies and planning for sustainability. The fact that agencies chose to invest in 3 years of CARE consultation after our implementation speaks to the program’s perceived value to agency leaders and their desire to sustain it.

Finally, our results say little about the mechanism of change responsible for the drop in incidents. Although consultants followed a standard progression of activities, implementation unfolded in unique ways at different agencies, and what was essential at one agency may not have helped another. More systematic inquiry is needed to better understand how CARE is able to take root and become integrated into the existing structures and priorities of an organization. Anglin has begun this work (see Holden et al. 2014), and future studies of the CARE approach will identify and measure key implementation drivers associated with successful outcomes.

Historically, the prevention field has focused disproportionately on individual-level intervention models designed to address internal risk and protective factors (Catalano et al. 2002). To achieve these goals, many have relied mostly on directive implementation models intended to deliver a set of more-or-less scripted, programmatic activities. As prevention work expands into ever more complex social settings (Hawe et al. 2009), program models are needed that depend less on standardized practices and more on creating a community of practice based on scientific knowledge and adaptive use of sound professional judgment. Similarly, organizational leaders need the frameworks and sustained guidance for how to support these capacities within the workforce. The current study illustrates a contextual approach that prevents dangerous incidents by providing a common set of principles that change how agency leaders and staff think and how they interact with residents and with each other. Approaches such as CARE may never replace individual-level program models, but they can create favorable conditions for effective prevention and health promotion.
